# Supplementary material for: Single Cell Transcriptomic and Chromatin Profiles Suggest Layer Vb Is the Only Layer With Shared Excitatory Cell Types in the Medial and Lateral Entorhinal Cortex
Source: Front Neural Circuits. 2022 Jan 26;15:806154. doi: 10.3389/fncir.2021.806154 (PMC8826650; doi:10.3389/fncir.2021.806154)
Supplement: Supplementary file 14 [file Data_Sheet_1.ZIP › 19/knownResults.html]

19 - Homer Known Motif Enrichment Results


# Homer Known Motif Enrichment Results (19)

Homer *de novo* Motif Results  
Gene Ontology Enrichment Results  
Known Motif Enrichment Results (txt file)  
Total Target Sequences = 1000, Total Background Sequences = 48608

|  |  |  |  |  |  |  |  |  |  |  |  |
| --- | --- | --- | --- | --- | --- | --- | --- | --- | --- | --- | --- |
| Rank | Motif | Name | P-value | log P-pvalue | q-value (Benjamini) | # Target Sequences with Motif | % of Targets Sequences with Motif | # Background Sequences with Motif | % of Background Sequences with Motif | Motif File | SVG |
| 1 | T C G A T G A C G T A C C G T A C A G T T G A C A C G T A C T G A G C T A G C T | NeuroG2(bHLH)/Fibroblast-NeuroG2-ChIP-Seq(GSE75910)/Homer | 1e-34 | -7.907e+01 | 0.0000 | 384.0 | 38.40% | 10324.3 | 21.24% | motif file (matrix) | svg |
| 2 | C T G A T G A C T G A C C G T A A C G T T G A C A G C T C T A G A C G T G A C T | Olig2(bHLH)/Neuron-Olig2-ChIP-Seq(GSE30882)/Homer | 1e-34 | -7.860e+01 | 0.0000 | 474.0 | 47.40% | 14071.5 | 28.95% | motif file (matrix) | svg |
| 3 | T C A G A C G T T C G A T A G C A G T C C G T A A C T G G T A C A C G T A C T G A T C G A G T C | Atoh1(bHLH)/Cerebellum-Atoh1-ChIP-Seq(GSE22111)/Homer | 1e-29 | -6.735e+01 | 0.0000 | 271.0 | 27.10% | 6553.3 | 13.48% | motif file (matrix) | svg |
| 4 | T A C G T G C A A G T C C G T A A C G T T G A C A C G T A C T G A C T G G C A T | TCF4(bHLH)/SHSY5Y-TCF4-ChIP-Seq(GSE96915)/Homer | 1e-26 | -6.179e+01 | 0.0000 | 363.0 | 36.30% | 10353.8 | 21.30% | motif file (matrix) | svg |
| 5 | C T A G T C G A T G A C A G T C C G T A A C T G G T A C A C G T A C T G A C T G | BHLHA15(bHLH)/NIH3T3-BHLHB8.HA-ChIP-Seq(GSE119782)/Homer | 1e-26 | -6.109e+01 | 0.0000 | 333.0 | 33.30% | 9192.1 | 18.91% | motif file (matrix) | svg |
| 6 | T C A G T G A C G T A C C G T A A C G T T G A C A C G T T C A G A G C T G A C T | NeuroD1(bHLH)/Islet-NeuroD1-ChIP-Seq(GSE30298)/Homer | 1e-25 | -5.915e+01 | 0.0000 | 217.0 | 21.70% | 4970.5 | 10.23% | motif file (matrix) | svg |
| 7 | T G A C A G T C C G T A A C T G G T A C A C G T A C T G A C G T G A C T G A T C | Twist2(bHLH)/Myoblast-Twist2.Ty1-ChIP-Seq(GSE127998)/Homer | 1e-23 | -5.433e+01 | 0.0000 | 390.0 | 39.00% | 11897.2 | 24.48% | motif file (matrix) | svg |
| 8 | C A T G A G T C G A C T C G T A C G A T G C A T G A C T G C A T C G A T C T A G C A T G T G A C | Mef2b(MADS)/HEK293-Mef2b.V5-ChIP-Seq(GSE67450)/Homer | 1e-20 | -4.805e+01 | 0.0000 | 274.0 | 27.40% | 7573.8 | 15.58% | motif file (matrix) | svg |
| 9 | C A T G G T A C G A C T G C T A C G T A C G T A C G T A G C T A G A C T C T G A T C A G G T A C | Mef2c(MADS)/GM12878-Mef2c-ChIP-Seq(GSE32465)/Homer | 1e-14 | -3.280e+01 | 0.0000 | 167.0 | 16.70% | 4349.6 | 8.95% | motif file (matrix) | svg |
| 10 | A C T G C T A G T C G A C G A T C A T G G C T A A T C G C G A T G T A C G C T A A G C T G T A C | Fra1(bZIP)/BT549-Fra1-ChIP-Seq(GSE46166)/Homer | 1e-13 | -3.190e+01 | 0.0000 | 144.0 | 14.40% | 3567.9 | 7.34% | motif file (matrix) | svg |
| 11 | C T A G A G C T G A C T C A T G A G T C A G T C G T C A C A G T C T A G T C A G G T A C C T G A T C G A G A T C T G A C | Rfx2(HTH)/LoVo-RFX2-ChIP-Seq(GSE49402)/Homer | 1e-13 | -3.168e+01 | 0.0000 | 39.0 | 3.90% | 416.5 | 0.86% | motif file (matrix) | svg |
| 12 | C T A G T C G A A C G T A C T G C G T A A T G C A C G T G T A C C G T A A G C T G A T C G T A C | Atf3(bZIP)/GBM-ATF3-ChIP-Seq(GSE33912)/Homer | 1e-13 | -3.142e+01 | 0.0000 | 161.0 | 16.10% | 4200.1 | 8.64% | motif file (matrix) | svg |
| 13 | A C T G C A T G T C G A A C G T A C T G C G T A A T C G A C G T G T A C C G T A G A C T A G T C | Fos(bZIP)/TSC-Fos-ChIP-Seq(GSE110950)/Homer | 1e-13 | -3.079e+01 | 0.0000 | 148.0 | 14.80% | 3762.9 | 7.74% | motif file (matrix) | svg |
| 14 | C T G A T C A G G T A C G C T A A C T G T G A C G C A T C A T G | SCL(bHLH)/HPC7-Scl-ChIP-Seq(GSE13511)/Homer | 1e-13 | -3.038e+01 | 0.0000 | 671.0 | 67.10% | 27002.7 | 55.56% | motif file (matrix) | svg |
| 15 | A G T C A T C G C T A G A G C T G A C T C T A G A G T C A G T C G C T A C A G T T C A G T C A G G A T C C T G A T C G A G A T C | RFX(HTH)/K562-RFX3-ChIP-Seq(SRA012198)/Homer | 1e-13 | -3.024e+01 | 0.0000 | 35.0 | 3.50% | 352.7 | 0.73% | motif file (matrix) | svg |
| 16 | C T A G A G T C T A C G T A C G T G A C C G T A A C T G T A G C G C A T C A T G A T G C A G C T | Ascl1(bHLH)/NeuralTubes-Ascl1-ChIP-Seq(GSE55840)/Homer | 1e-12 | -2.939e+01 | 0.0000 | 263.0 | 26.30% | 8298.4 | 17.08% | motif file (matrix) | svg |
| 17 | T C G A A G T C C G T A A T C G T A G C A C G T A C T G A G C T A C G T A G T C | Ptf1a(bHLH)/Panc1-Ptf1a-ChIP-Seq(GSE47459)/Homer | 1e-12 | -2.923e+01 | 0.0000 | 431.0 | 43.10% | 15592.7 | 32.08% | motif file (matrix) | svg |
| 18 | C A G T T G C A A C G T A C T G C G T A A T C G C G A T T G A C C G T A A C G T | BATF(bZIP)/Th17-BATF-ChIP-Seq(GSE39756)/Homer | 1e-12 | -2.907e+01 | 0.0000 | 161.0 | 16.10% | 4323.0 | 8.90% | motif file (matrix) | svg |
| 19 | C T A G T C G A G C A T C A T G G C T A T A G C C G A T G T A C C T G A A G C T | JunB(bZIP)/DendriticCells-Junb-ChIP-Seq(GSE36099)/Homer | 1e-12 | -2.818e+01 | 0.0000 | 134.0 | 13.40% | 3389.5 | 6.97% | motif file (matrix) | svg |
| 20 | C A T G C T A G T C G A A C G T A C T G C G T A T A G C C G A T T G A C C G T A A G C T G A T C | Fra2(bZIP)/Striatum-Fra2-ChIP-Seq(GSE43429)/Homer | 1e-11 | -2.684e+01 | 0.0000 | 116.0 | 11.60% | 2819.3 | 5.80% | motif file (matrix) | svg |
| 21 | A G T C G A C T C A G T A C T G C T A G T G A C G C T A A T G C G C A T A T C G C G A T A C T G G A T C G T A C G T C A C T G A | NF1(CTF)/LNCAP-NF1-ChIP-Seq(Unpublished)/Homer | 1e-11 | -2.538e+01 | 0.0000 | 81.0 | 8.10% | 1710.3 | 3.52% | motif file (matrix) | svg |
| 22 | T C G A A C G T C A T G G C T A T A G C C G A T G T A C G C T A A C G T A T G C | AP-1(bZIP)/ThioMac-PU.1-ChIP-Seq(GSE21512)/Homer | 1e-10 | -2.504e+01 | 0.0000 | 165.0 | 16.50% | 4705.8 | 9.68% | motif file (matrix) | svg |
| 23 | C T A G T C G A A C G T A C T G C G T A T A G C C G A T G T A C C G T A A G C T G A T C G T A C | Jun-AP1(bZIP)/K562-cJun-ChIP-Seq(GSE31477)/Homer | 1e-10 | -2.452e+01 | 0.0000 | 65.0 | 6.50% | 1243.2 | 2.56% | motif file (matrix) | svg |
| 24 | G T A C G A C T C G T A C T G A T C G A C G T A G C T A C A G T C T G A T A C G | Mef2a(MADS)/HL1-Mef2a.biotin-ChIP-Seq(GSE21529)/Homer | 1e-9 | -2.289e+01 | 0.0000 | 136.0 | 13.60% | 3740.5 | 7.70% | motif file (matrix) | svg |
| 25 | C A T G C T A G A G C T G A C T C A T G A G T C G A T C G C T A C G A T C T A G T C A G G T A C C T G A T C G A | X-box(HTH)/NPC-H3K4me1-ChIP-Seq(GSE16256)/Homer | 1e-9 | -2.180e+01 | 0.0000 | 41.0 | 4.10% | 634.6 | 1.31% | motif file (matrix) | svg |
| 26 | C A T G A G T C A G C T C G T A C G A T C G A T G C A T G C A T C G A T C T G A C A T G T G A C | Mef2d(MADS)/Retina-Mef2d-ChIP-Seq(GSE61391)/Homer | 1e-9 | -2.169e+01 | 0.0000 | 75.0 | 7.50% | 1653.2 | 3.40% | motif file (matrix) | svg |
| 27 | C T A G T C G A C G A T A C T G C G T A T A C G A G C T T G A C G C T A A C G T G A T C T A G C | Fosl2(bZIP)/3T3L1-Fosl2-ChIP-Seq(GSE56872)/Homer | 1e-9 | -2.084e+01 | 0.0000 | 77.0 | 7.70% | 1752.9 | 3.61% | motif file (matrix) | svg |
| 28 | T G A C A T G C C G T A A T C G A T G C C A G T C A T G A C T G A G T C G T A C | HEB(bHLH)/mES-Heb-ChIP-Seq(GSE53233)/Homer | 1e-8 | -1.846e+01 | 0.0000 | 286.0 | 28.60% | 10224.3 | 21.04% | motif file (matrix) | svg |
| 29 | A G C T A C G T A C T G A T G C A G T C C G T A C T G A T A C G | NF1-halfsite(CTF)/LNCaP-NF1-ChIP-Seq(Unpublished)/Homer | 1e-6 | -1.525e+01 | 0.0000 | 271.0 | 27.10% | 9919.9 | 20.41% | motif file (matrix) | svg |
| 30 | A C T G T C A G A G C T G A C T C A T G A G T C A G T C G C T A C G A T C T A G T C A G G T A C C T G A T C G A | Rfx1(HTH)/NPC-H3K4me1-ChIP-Seq(GSE16256)/Homer | 1e-6 | -1.515e+01 | 0.0000 | 48.0 | 4.80% | 1028.9 | 2.12% | motif file (matrix) | svg |
| 31 | T C A G A G C T A C G T A C G T G T A C G A T C C G T A C T A G C A T G G T C A C G T A T C G A | STAT4(Stat)/CD4-Stat4-ChIP-Seq(GSE22104)/Homer | 1e-6 | -1.443e+01 | 0.0000 | 212.0 | 21.20% | 7458.2 | 15.35% | motif file (matrix) | svg |
| 32 | T A C G C T A G T A C G A G T C C G T A A G T C A G T C A C G T A C T G A G T C G A T C T A G C | Slug(Zf)/Mesoderm-Snai2-ChIP-Seq(GSE61475)/Homer | 1e-5 | -1.261e+01 | 0.0000 | 91.0 | 9.10% | 2686.8 | 5.53% | motif file (matrix) | svg |
| 33 | A T G C A G T C G T A C A G C T T C G A C T A G G A T C C T G A G T C A A G T C G C T A T C A G | Rfx5(HTH)/GM12878-Rfx5-ChIP-Seq(GSE31477)/Homer | 1e-5 | -1.257e+01 | 0.0000 | 90.0 | 9.00% | 2651.8 | 5.46% | motif file (matrix) | svg |
| 34 | T C G A T A G C G T C A A C T G A C T G C G T A C G T A C T A G A G C T T C A G | ERG(ETS)/VCaP-ERG-ChIP-Seq(GSE14097)/Homer | 1e-5 | -1.180e+01 | 0.0001 | 270.0 | 27.00% | 10301.6 | 21.20% | motif file (matrix) | svg |
| 35 | A G T C C G A T A C T G A T C G T G A C G C T A C A T G A T C G T G A C C G A T A C T G T A G C G T A C G T C A | Tlx?(NR)/NPC-H3K4me1-ChIP-Seq(GSE16256)/Homer | 1e-5 | -1.155e+01 | 0.0001 | 73.0 | 7.30% | 2076.4 | 4.27% | motif file (matrix) | svg |
| 36 | A T G C T C G A A G T C A G C T A C G T G T A C A G T C G C T A C T A G C A T G G T C A C T G A T C A G A G T C | Stat3+il21(Stat)/CD4-Stat3-ChIP-Seq(GSE19198)/Homer | 1e-4 | -1.086e+01 | 0.0002 | 140.0 | 14.00% | 4795.3 | 9.87% | motif file (matrix) | svg |
| 37 | G A C T C G A T C T G A G T C A G A C T C G A T T C G A C G T A G C T A G C T A T G A C G T A C C G T A A C T G T G C A C G A T A C T G A C G T | Pitx1:Ebox(Homeobox,bHLH)/Hindlimb-Pitx1-ChIP-Seq(GSE41591)/Homer | 1e-4 | -1.055e+01 | 0.0003 | 63.0 | 6.30% | 1766.2 | 3.63% | motif file (matrix) | svg |
| 38 | A G T C A C G T A C T G A G C T A C G T A C G T G T C A A G T C | Foxo1(Forkhead)/RAW-Foxo1-ChIP-Seq(Fan\_et\_al.)/Homer | 1e-4 | -1.035e+01 | 0.0004 | 351.0 | 35.10% | 14193.3 | 29.21% | motif file (matrix) | svg |
| 39 | C G T A T G A C T C G A A G T C C G T A A T C G A T G C A C G T A C T G A G T C | E2A(bHLH)/proBcell-E2A-ChIP-Seq(GSE21978)/Homer | 1e-4 | -1.026e+01 | 0.0004 | 199.0 | 19.90% | 7375.9 | 15.18% | motif file (matrix) | svg |
| 40 | G A C T C T A G A T G C A G T C G T C A T A C G A T G C A T C G | HIC1(Zf)/Treg-ZBTB29-ChIP-Seq(GSE99889)/Homer | 1e-4 | -1.005e+01 | 0.0005 | 281.0 | 28.10% | 11044.3 | 22.73% | motif file (matrix) | svg |
| 41 | A G C T T C G A G T A C T C G A A T G C A T G C G C A T A T C G A G T C A G C T | Snail1(Zf)/LS174T-SNAIL1.HA-ChIP-Seq(GSE127183)/Homer | 1e-4 | -9.868e+00 | 0.0006 | 129.0 | 12.90% | 4438.4 | 9.13% | motif file (matrix) | svg |
| 42 | C A G T T C A G G A T C A C T G A C G T C T A G A C T G A C T G G A C T C T A G | Egr1(Zf)/K562-Egr1-ChIP-Seq(GSE32465)/Homer | 1e-4 | -9.834e+00 | 0.0006 | 80.0 | 8.00% | 2462.0 | 5.07% | motif file (matrix) | svg |
| 43 | T C G A A G C T A C G T A C G T A G T C A G T C A C G T A T C G G A C T A T C G | EWS:ERG-fusion(ETS)/CADO\_ES1-EWS:ERG-ChIP-Seq(SRA014231)/Homer | 1e-4 | -9.818e+00 | 0.0006 | 155.0 | 15.50% | 5536.3 | 11.39% | motif file (matrix) | svg |
| 44 | A G T C G A C T C A G T G T A C A G T C A T C G T C A G A C T G G T C A C G T A | Stat3(Stat)/mES-Stat3-ChIP-Seq(GSE11431)/Homer | 1e-4 | -9.579e+00 | 0.0007 | 95.0 | 9.50% | 3076.5 | 6.33% | motif file (matrix) | svg |
| 45 | C T G A T A G C T G A C T C A G C T A G G T C A C G T A T C A G A G C T T C A G | ETV4(ETS)/HepG2-ETV4-ChIP-Seq(ENCODE)/Homer | 1e-3 | -9.116e+00 | 0.0011 | 160.0 | 16.00% | 5832.6 | 12.00% | motif file (matrix) | svg |
| 46 | C G T A A C G T A C G T A C G T A C G T A G T C A G T C C T G A A G C T A G C T | NFAT(RHD)/Jurkat-NFATC1-ChIP-Seq(Jolma\_et\_al.)/Homer | 1e-3 | -9.080e+00 | 0.0011 | 201.0 | 20.10% | 7618.6 | 15.68% | motif file (matrix) | svg |
| 47 | T G C A C T G A A T G C G T C A A C T G A C T G C G T A C G T A C T A G A G C T | Ets1-distal(ETS)/CD4+-PolII-ChIP-Seq(Barski\_et\_al.)/Homer | 1e-3 | -8.814e+00 | 0.0014 | 67.0 | 6.70% | 2034.2 | 4.19% | motif file (matrix) | svg |
| 48 | C A G T T C A G A G C T G A C T A C G T A G T C G A T C G A C T C T G A A C T G G A T C C G T A C T G A A G T C G T A C | Rfx6(HTH)/Min6b1-Rfx6.HA-ChIP-Seq(GSE62844)/Homer | 1e-3 | -8.797e+00 | 0.0014 | 173.0 | 17.30% | 6433.7 | 13.24% | motif file (matrix) | svg |
| 49 | T C G A T C G A T A G C G T A C T C A G T A C G C G T A C G T A T C A G A G C T | GABPA(ETS)/Jurkat-GABPa-ChIP-Seq(GSE17954)/Homer | 1e-3 | -8.344e+00 | 0.0021 | 139.0 | 13.90% | 5027.3 | 10.34% | motif file (matrix) | svg |
| 50 | A T G C A G T C C T G A A G T C C G A T A C G T A G T C A G T C A C G T A T C G G A C T A C G T | Etv2(ETS)/ES-ER71-ChIP-Seq(GSE59402)/Homer | 1e-3 | -8.285e+00 | 0.0022 | 159.0 | 15.90% | 5893.5 | 12.13% | motif file (matrix) | svg |
| 51 | C G A T T A C G T G A C G A C T C A T G C G T A T A C G A C G T G T A C C T G A | Bach2(bZIP)/OCILy7-Bach2-ChIP-Seq(GSE44420)/Homer | 1e-3 | -8.268e+00 | 0.0022 | 38.0 | 3.80% | 989.9 | 2.04% | motif file (matrix) | svg |
| 52 | C G A T T G C A T G C A G A T C C G T A A C T G T G A C G A C T C A T G A C T G | Tcf21(bHLH)/ArterySmoothMuscle-Tcf21-ChIP-Seq(GSE61369)/Homer | 1e-3 | -8.124e+00 | 0.0025 | 141.0 | 14.10% | 5139.0 | 10.57% | motif file (matrix) | svg |
| 53 | C A T G A C T G C T A G T C G A T C G A T C G A T C G A T C A G T C A G T C A G T G A C T G A C C G T A A C T G T G C A C G A T A C T G | RBPJ:Ebox(?,bHLH)/Panc1-Rbpj1-ChIP-Seq(GSE47459)/Homer | 1e-3 | -7.636e+00 | 0.0040 | 62.0 | 6.20% | 1929.6 | 3.97% | motif file (matrix) | svg |
| 54 | T A C G T A C G C T A G T C A G A G T C C G T A A T C G A T G C A C G T A C T G A G T C G A C T | Ascl2(bHLH)/ESC-Ascl2-ChIP-Seq(GSE97712)/Homer | 1e-3 | -7.520e+00 | 0.0044 | 147.0 | 14.70% | 5474.8 | 11.27% | motif file (matrix) | svg |
| 55 | T C G A C T G A T A G C T G A C T C A G T C A G C G T A C G T A T C A G A G C T | ETV1(ETS)/GIST48-ETV1-ChIP-Seq(GSE22441)/Homer | 1e-3 | -7.508e+00 | 0.0044 | 213.0 | 21.30% | 8383.7 | 17.25% | motif file (matrix) | svg |
| 56 | C T G A A T G C C G T A A C G T A G T C A G T C A C G T A C T G A T C G G C A T | SPDEF(ETS)/VCaP-SPDEF-ChIP-Seq(SRA014231)/Homer | 1e-3 | -7.319e+00 | 0.0052 | 178.0 | 17.80% | 6857.0 | 14.11% | motif file (matrix) | svg |
| 57 | A T G C C T G A G A C T A C G T A C G T G T A C G A T C C G A T C T A G C A T G C G T A C G T A C T G A G A C T | STAT1(Stat)/HelaS3-STAT1-ChIP-Seq(GSE12782)/Homer | 1e-3 | -7.081e+00 | 0.0065 | 72.0 | 7.20% | 2373.9 | 4.88% | motif file (matrix) | svg |
| 58 | A G T C C T G A A G T C C G A T C A G T G A T C A T G C A C T G A T C G G A C T | Fli1(ETS)/CD8-FLI-ChIP-Seq(GSE20898)/Homer | 1e-3 | -6.978e+00 | 0.0071 | 163.0 | 16.30% | 6247.2 | 12.85% | motif file (matrix) | svg |
| 59 | C T G A A G T C C G A T A G C T A T G C G T A C A C G T A T C G C A G T G C A T | Elf4(ETS)/BMDM-Elf4-ChIP-Seq(GSE88699)/Homer | 1e-2 | -6.757e+00 | 0.0087 | 166.0 | 16.60% | 6412.3 | 13.19% | motif file (matrix) | svg |
| 60 | A T G C T G C A A G T C C G T A A G T C A G T C A C G T A C T G A T G C G T C A | E2A(bHLH),near\_PU.1/Bcell-PU.1-ChIP-Seq(GSE21512)/Homer | 1e-2 | -6.554e+00 | 0.0105 | 197.0 | 19.70% | 7823.8 | 16.10% | motif file (matrix) | svg |
| 61 | T C A G G A C T C A G T C T G A A G C T C T A G G A C T T G C A C T G A A G T C | HLF(bZIP)/HSC-HLF.Flag-ChIP-Seq(GSE69817)/Homer | 1e-2 | -6.072e+00 | 0.0166 | 181.0 | 18.10% | 7189.0 | 14.79% | motif file (matrix) | svg |
| 62 | C T G A A C G T A C G T A C G T A G T C G A C T C G A T C T G A A C T G C G T A C G T A T C G A | STAT5(Stat)/mCD4+-Stat5-ChIP-Seq(GSE12346)/Homer | 1e-2 | -5.886e+00 | 0.0197 | 78.0 | 7.80% | 2738.0 | 5.63% | motif file (matrix) | svg |
| 63 | G T A C G A T C C A G T A G T C A G T C A G T C T G C A G A T C C T G A A T G C G T C A A C G T | WT1(Zf)/Kidney-WT1-ChIP-Seq(GSE90016)/Homer | 1e-2 | -5.601e+00 | 0.0258 | 64.0 | 6.40% | 2189.5 | 4.51% | motif file (matrix) | svg |
| 64 | T A G C T C A G C A T G G C A T A G C T C G A T A T G C C G T A C G T A G T C A | CHR(?)/Hela-CellCycle-Expression/Homer | 1e-2 | -5.462e+00 | 0.0292 | 145.0 | 14.50% | 5687.3 | 11.70% | motif file (matrix) | svg |
| 65 | C A T G A C G T A G T C G A T C G A T C G A T C G C T A C T A G C T A G C T A G T C A G T C G A | EBF1(EBF)/Near-E2A-ChIP-Seq(GSE21512)/Homer | 1e-2 | -5.335e+00 | 0.0326 | 113.0 | 11.30% | 4301.9 | 8.85% | motif file (matrix) | svg |
| 66 | T C G A A C G T A C G T C T G A G A T C T C A G G A C T G T C A C G T A A G C T G T C A C T A G A G C T A C G T T C G A | NFIL3(bZIP)/HepG2-NFIL3-ChIP-Seq(Encode)/Homer | 1e-2 | -5.322e+00 | 0.0326 | 131.0 | 13.10% | 5091.2 | 10.48% | motif file (matrix) | svg |
| 67 | C G T A T A G C T A G C T G C A A C T G C T A G C G T A C G T A T C A G G A C T | EHF(ETS)/LoVo-EHF-ChIP-Seq(GSE49402)/Homer | 1e-2 | -5.137e+00 | 0.0386 | 224.0 | 22.40% | 9312.0 | 19.16% | motif file (matrix) | svg |
| 68 | A T G C A G T C G C T A C G A T C G T A G C A T G C T A C G A T C T A G C A T G T G A C G T C A | CArG(MADS)/PUER-Srf-ChIP-Seq(Sullivan\_et\_al.)/Homer | 1e-2 | -5.028e+00 | 0.0424 | 64.0 | 6.40% | 2249.2 | 4.63% | motif file (matrix) | svg |
| 69 | T C G A A C T G A C T G C G T A C G T A T C G A A G T C C T G A A T C G G T A C G C A T C A T G | ETS:E-box(ETS,bHLH)/HPC7-Scl-ChIP-Seq(GSE22178)/Homer | 1e-2 | -4.955e+00 | 0.0450 | 19.0 | 1.90% | 487.8 | 1.00% | motif file (matrix) | svg |
| 70 | C T G A A G C T A C G T A C G T A G T C G A C T G A C T C T G A C T G A C T A G C G T A C G T A | STAT6(Stat)/CD4-Stat6-ChIP-Seq(GSE22104)/Homer | 1e-2 | -4.756e+00 | 0.0540 | 116.0 | 11.60% | 4520.4 | 9.30% | motif file (matrix) | svg |
| 71 | T C G A T A G C T G C A A C T G A C T G C G T A C G T A C T A G G A C T T A C G | ETS1(ETS)/Jurkat-ETS1-ChIP-Seq(GSE17954)/Homer | 1e-2 | -4.714e+00 | 0.0556 | 160.0 | 16.00% | 6488.5 | 13.35% | motif file (matrix) | svg |
| 72 | C G A T C T A G C T G A A T G C C T G A T C G A C G T A C T G A T C G A T A G C A G T C C G T A A C T G T C G A A T G C | Hand2(bHLH)/Mesoderm-Hand2-ChIP-Seq(GSE61475)/Homer | 1e-2 | -4.666e+00 | 0.0575 | 82.0 | 8.20% | 3054.7 | 6.29% | motif file (matrix) | svg |
